# Minimum ten-year outcome of a triple-tapered femoral stem implanted with line-to-line cementing technique

**DOI:** 10.1186/s12891-021-04484-2

**Published:** 2021-06-30

**Authors:** Hirotsugu Ohashi, Satoshi Iida, Izumi Minato

**Affiliations:** 1 Department of Orthopaedic Surgery, Osaka Saiseikai Nakatsu Hospital, 2-10-39 Shibata, Kita-ku, Osaka, 530-0012 Japan; 2 Department of Orthopaedic Surgery, Matsudo City General Hospital, 933-1 Sendabori, Matsudo, Chiba, 270-2296 Japan; 3 Department of Orthopaedic Surgery, Niigata Rinko Hospital, 1-114-3 Momoyama-cho, higashi-ku, Niigata, 950-8725 Japan

**Keywords:** Total hip arthroplasty, Triple-tapered polished femoral stem, Line-to-line cementing technique, Subsidence

## Abstract

**Background:**

A triple-tapered polished femoral stem was implanted with line-to-line cementing technique. The purpose of this study was to determine the survivorship, loosening rate, stem subsidence, radiologic changes and clinical outcomes in the minimum 10-year follow-up.

**Methods:**

This was a retrospective study done in three institutes. Finally, 118 hips in 97 patients could be followed-up at the mean follow-up period of 126.3 months. The survivorship, radiological and clinical outcomes were investigated.

**Results:**

Radiologically, 107 hips (90.7%) were categorized to Barrack cementing grade A, and 108 stems (91.5%) were inserted in neutral position. All hips were not loose and were not revised due to any reason. Survival with revision for any reason as the endpoint was 100% after 10 years. At the last follow-up, the mean subsidence was 0.43 mm, and the subsidence was less than 1 mm in 110 hips (93.2%).

JOA hip score improved from 42.7 ± 8.9 points preoperatively to 92.8 ± 6.8 points at the last follow-up. No patient complained thigh pain.

**Conclusions:**

Line-to-line cementing technique with use of a triple-tapered polished stem was effective to achieve good cementation quality and centralization of the stem. The subsidence was small, and the minimum 10-year results were excellent without any failures related to the stem.

**Trial registration:**

Retrospectively registered.

## Background

Since the cement fixation system of femoral stems for total hip arthroplasty (THA) was established in the 1960s, a variety of different concepts have been applied to the development of the femoral stems. In these concepts, main topics are stem design and cement mantle thickness.

In terms of stem design, cemented femoral stems can be broadly divided into two designs that achieve fixation as a composite beam and those that function as a taper-slip device [1]. Recent studies of polished tapered cemented stems have reported superior long-term clinical outcomes from the systematic review [2], and significant survival advantages in the registry data [3]. Originally, double-tapered polished stems, such as the Exeter stem and the CPT stem, were developed. Their long-term good clinical results have been previously reported [4–6], while the bone loss in the calcar region around double-tapered stem was concerning [7]. A triple-tapered cemented stem was designed with the intention of loading the proximal femur, thereby reducing proximal bone loss [8]. Buckland et al. [9] measured bone mineral density (BMD) around a triple-tapered stem. The marked loss in BMD occurred in zone 1 and 7 within 9 months postoperatively, while zones 6 and 7 showed a recovery in BMD between 9 and 24 months postoperatively, and zones 1 and 3 showed more delayed recovery in BMD at 18 months.

The second topic of cemented stem is cement mantle thickness. It is still unclear whether thickening the cement mantle leads to better outcomes. Many studies have reported good outcomes for a cement mantle thickness of at least 2 mm [10–13], while a favorable outcome with a thin cement mantle was also reported as ‘French paradox’ [14, 15]. Line-to-line cementing technique means preparation of the femoral canal using the largest possible broach and implantation of a stem with the same dimensions as the broach. It is reported that line-to-line cementing technique in human cadaver femora resulted in a mean thickness of cement of 3.1 mm, and the cement was directly supported by cortical bone or cortical bone with less than 1 mm of cancellous bone interposed in over 90% of thin cement mantle areas [16]. This technique also can achieve the pressurization of the cement into cancellous bone during insertion of the implant [17]. These results indicate that both polished tapered stem design and line-to-line cementing technique might bring about successful long-term outcomes.

The purpose of this study was to determine the survivorship, loosening rate, stem subsidence, radiologic changes and clinical outcomes in the minimum 10-year follow-up of a triple-tapered polished stem implanted with line-to-line cementing technique. These results were compared with those of the other polished tapered stems.

## Materials and methods

The research protocol of this retrospective study was in compliance with the Helsinki Declaration. The institutional review board of Osaka Saiseikai Nakatsu Hospital approved this study. Informed consent was obtained from all patients who participated in this study.

**Fig. 1:**
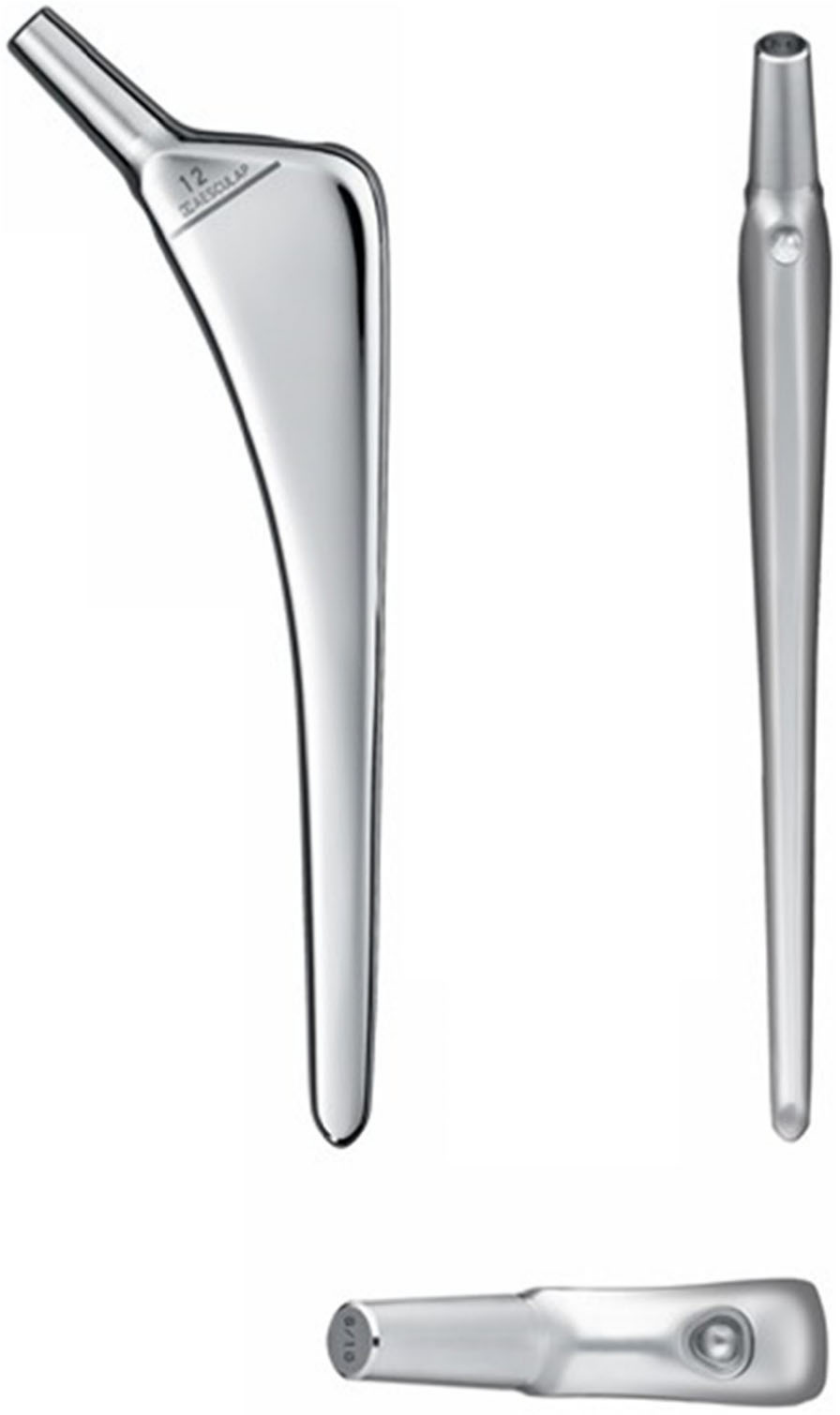
Three views of a triple-tapered polished cemented stem, Trilliance

**Fig. 2:**
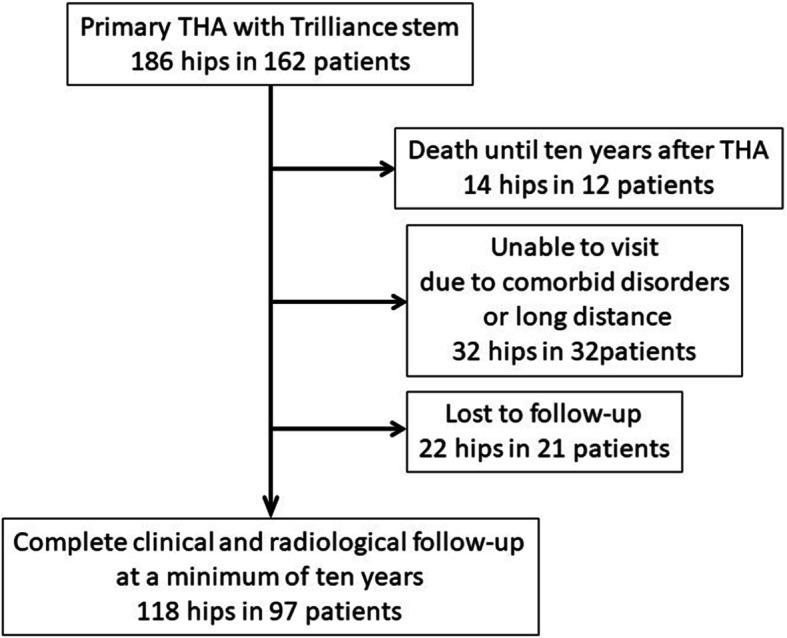
The flowchart of patients in this study

From February 2009 to October 2010, consecutive 186 hips in 162 patients underwent THA with a femoral stem (Trilliance, B. Braun Aesculap, Tuttlingen, Germany) fixed with line-to-line cementing technique. This stem was made of CoCr with a highly polished surface (Ra 0.01 mm) and a quadrangular section (Fig. 1). Twelve patients died, 14 patients could not visit due to the comorbid disorders, 18 patients could not visit due to long distance to the institutes, and 21 patients were lost to follow-up. Finally, 118 hips in 97 patients could be followed-up at longer than 10 years postoperatively (Fig. 2). The patients’ demographics, diagnosis, and surgical approaches were shown in Table 1. No patient received prior surgery to the hip. A type of cup and bearing materials were chosen according to the surgeons’ preference (Table 2). The heads used were 22, 26, 28 and 32 mm in diameter. Femoral canal was prepared using the largest possible broach, and bone plug or polyethylene cement plug was placed at 1 cm distal to the stem tip. After pulse lavage, vacuum mixed bone cement was introduced in a retrograde fashion. The femoral stem was inserted without a distal centralizer.

**Table 1:** Patient demographics, diagnosis, and surgical approaches

Demographics	
Number of hips	118 hips
Age at operation (mean ± SD, range)	61.2 ± 10.0 (20–84) years
Sex	
Males	18 hips
Females	100
Body weight (mean ± SD, range)	55.9 ± 9.6 (38–81) kg
BMI (mean ± SD, range)	23.3 ± 3.2 (17.4–33.6)
Follow-up period (mean ± SD, range)	126.3 ± 5.8 (120–139) months
Diagnosis	
Osteoarthritis	104 hips
*Primary osteoarthritis*	*12*
*Dysplastic osteoarthritis*	*92*
Idiopathic osteonecrosis of femoral head	9
Rheumatoid arthritis	2
Rapidly destructive coxarthritis	2
Miscellaneous	1
Approaches	
Direct anterior approach	97 hips
Direct lateral approach	20
Transtrochanteric approach	1

*BMI* Body mass index, *SD* Standard deviation

**Table 2:** Cups and types of articulation used in this study

Cup	
Plasmacup	68 hips
Contemporary	5
Triad HA	2
Trident	43
Articulation	
Ceramic / Ceramic	68 hips
Metal / Polyethylene	50

Clinical and radiological evaluation was undertaken preoperatively and at 3 weeks (baseline radiograph) and every year. At each follow-up visit, the patients had a physical examination by the operating surgeons and the functional results were evaluated using Japanese Orthopaedic Association hip score (JOA hip score, full mark = 100) [18]. Anteroposterior radiograph of the pelvis with the patient supine position was obtained, and all the radiographs were examined independently by the three authors. The cementing technique was assessed using the grading of Barrack et al. [19]. The alignment of the stem was referenced from the axial alignment of the femur, and the alignment was assumed to be neutral within 3 degrees from co-linearity. On the final radiographs, the presence and evolution of radiolucent lines and cortical thickening in any of the seven zones described by Gruen et al. [20] was evaluated. Loosening of the femoral component was defined according to the criteria of Harris et al. [21] which included subsidence of the stem greater than 3 mm, fracture of the cement mantle, and a complete radiolucent line greater than 2 mm or a radiolucent line in zone 1 greater than 2 mm in width. Periprosthetic cystic or scalloped lesions larger than 2 mm in diameter which had not been present on the immediate post-operative radiograph were defined as osteolysis. Subsidence of the stem was measured on magnified images calibrated using the known size of the femoral head. The resolution of the screen (RadiForce RX340, EIZO, Ishikawa, Japan) was 121 pixel per inch. The radiological landmarks were the greater trochanter, the proximolateral cement mantle and the shoulder of the prosthesis, as described by the Exeter group [22].

All data were collected and analyzed using the Microsoft Excel Software (Microsoft Corporation, Redmond, WA). Kaplan-Meier survival analyses (EZR, Saitama Medical Center, Jichi Medical University, Saitama, Japan) were used to evaluate the cumulative stem survivorship and performed for any reasons, and aseptic loosening.

## Results

**Fig. 3:**
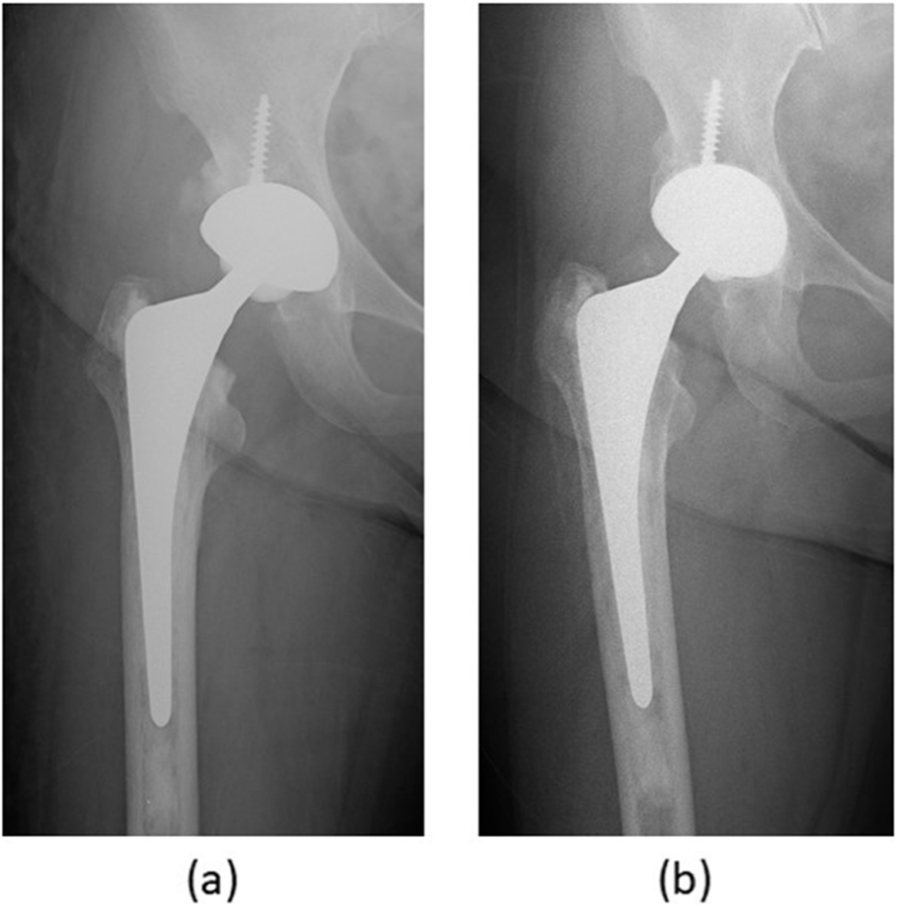
Post-operative anteroposterior radiographs of 62-year-old woman with dysplastic hip osteoarthritis showing (**a**) well-aligned stem cemented with line-to-line technique, (**b**) follow-up at 10 years without measurable subsidence of the stem

According to the Barrack cementing grade, 107 hips were categorized to grade A, 11 hips were categorized to grade B. Stem was inserted in neutral position in 108 hips, in valgus in 7 hips, and in varus in 3 hips. Radiolucent lines and osteolysis were not observed in any hips. According to the criteria of Harris et al. [21], all stems were not loose, and no stem and no cup were revised due to any reason (Fig. 3). Cortical thickening was observed in one hip at zones 3 and 5. Fracture of the cement mantle was not observed. No post-operative complication occurred, such as deep venous thrombosis, and heterotopic ossification.

**Fig. 4:**
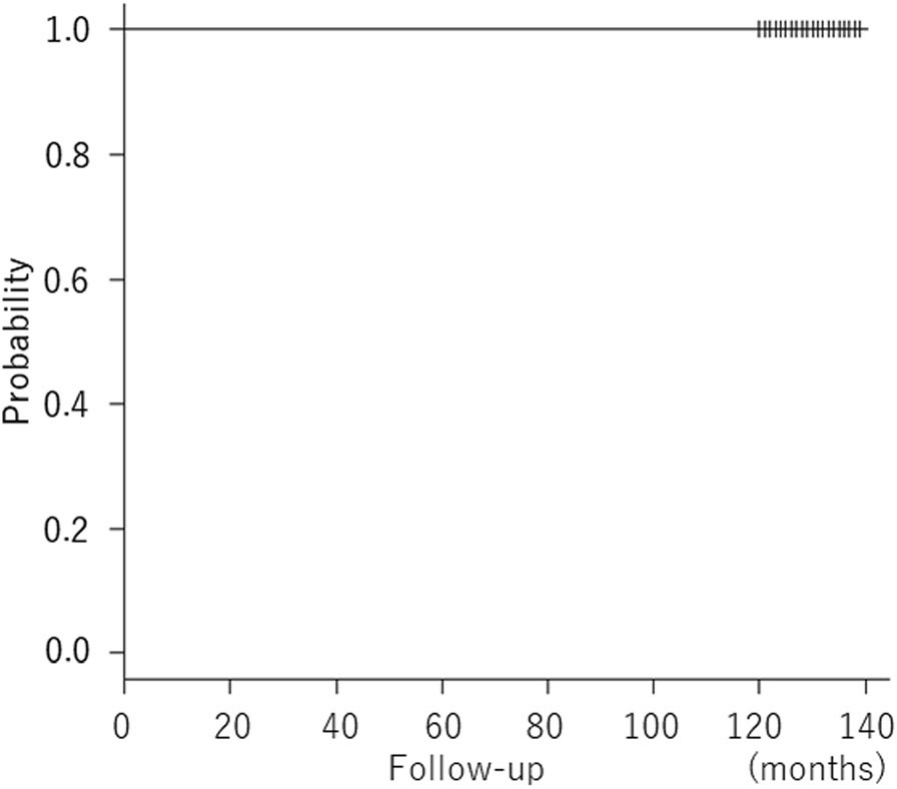
Kaplan Meier survival curve of stem revision for any reason as the endpoint

Survival with stem revision for any reason as the endpoint was 100% (Fig. 4). At the last follow-up, the mean subsidence was 0.43 ± 0.38 mm (0 mm to 1.9 mm). In most of the hips, the subsidence was less than 1 mm (Table 3).

**Table 3:** Number of hips in every 0.5 mm for subsidence

Subsidence	Frequency	Cumulative frequency
0 mm	28 hips	28 hips
0.5 mm > S > 0 mm	46	74
1 mm > S ≧ 0.5 mm	36	110
1.5 mm > S ≧ 1 mm	4	114
2 mm > S ≧ 1.5 mm	4	118

*S* Subsidence

JOA hip score improved from 42.7 ± 8.9 points preoperatively to 92.8 ± 6.8 points at the last follow-up. No patient complained thigh pain.

## Discussion

This is a first report about the long-term results of a triple-tapered stem implanted with line-to-line cementing technique. In this study, survival with revision for any reasons as the endpoint was 100%. The most widely used polished tapered femoral component, Exeter stem, showed that the survival rate for all-cause revision of the stem was 96.8%, and that for aseptic loosening as the endpoint was 100% at 13.5 years [5]. From the report of C-stem, there were no revision for stem loosening but two stems were revised for fracture at a mean follow-up of 13 years [23]. CMK stem, implanted with line-to-line cementing technique, marked the cumulative survival rate with revision of either component for any reason as endpoint of 90.5% at 17 years [15]. Our long-term results would be comparable to those of polished tapered stems.

Line-to-line cementing technique has some advantages. It can achieve the pressurization of the cement into cancellous bone during insertion of the implant [16]. While grade A cementation quality described by Barrack was recognized in 49.6 and 73.6% when using the Exeter stems [5, 24], grade A was achieved in 90.7% in our study. Other advantage is centralization of the stem. Scheerlinck et al. reported line-to-line stems without a distal centralizer were better aligned than undersized stems fitted with a centralizer [16]. Cortical point contact during stem insertion may improve alignment of the stem into the proximal medullary cavity. In our study, 91.5% was inserted in neutral position, and the CMK stem that was also implanted with line-to-line cementing technique, was in a neutral position in 80.5% [15]. In contrast, 65.3% was inserted within 2 degrees of varus or valgus in the Exeter stem [24], and 61.7% was in a neutral position in the C-stem [25].

The mean subsidence was 0.43 mm at the mean follow-up period of 126.9 months. It was reported that the mean subsidence of Exeter stem was 1.2 mm at a minimum of 10-year follow-up [5]. The subsidence of C-stem AMT was reported to be 1.28 mm at 2 years [26]. A hollow polymethyl-methacrylate centralizer was placed on the tip of these stems. The use of centralizer and thick cement mantle might allow these stems for subsidence. Subsidence remains a fundamental principle of the design of taper-slip stem, which produces tensile hoop forces in the cement and compressive stress at the cement-bone interface. On the other hand, the mean subsidence was 0.63 mm in CMK stem, that had no centralizer and was implanted with line-to-line cementing technique [15].

Cortical thickening was observed in one hip (0.8%) in our study. The incidence of cortical thickening was reported from 0.5 to 40.2% [4, 15, 27]. The rate of cortical thickening was lower compared to those reported results. Cortical hypertrophy might reflect the mechanical conditions around cemented femoral components, however, this was not matched to poor outcome, and its clinical relevance is still unclear.

The first limitation of the study is the uncontrolled retrospective study, and there was no control group. Thus, the results were compared with those of the other polished tapered stems. The Exeter stem and the CPT stem were double-tapered stem, and the C-stem was triple-tapered stem. These stems were fixed using distal centralizer with keeping cement mantle. The CMK stem was also double-tapered stem and the line-to-line cementing technique was applied. Secondary, the demographics of our patients were not similar to those in the previous studies. The main diagnosis of our patients was dysplastic osteoarthritis, and the majority of our patients were Japanese female. The body weight and BMI of our patients were lower. This bias might affect our results. Thirdly, the sample size was small and the follow-up period was not enough to compare our results with reported long-term results. For predicting the long-term survival of this femoral stem, we have to continue to investigate the clinical and radiological outcomes.

In conclusion, line-to-line cementing technique with use of a triple-tapered polished stem was effective to achieve good cementation quality and centralization of the stem. The subsidence was smaller than that of well-established polished tapered stems. The minimum 10-year results were excellent without any failures related to the stem. Further follow-up is needed to compare with results with longer-term follow-up.

## Data Availability

The datasets used and anlysed during the current study are available from the corresponding author on reasonable request.

## Abbreviations

THA: Total hip arthroplasty
BMD: Bone mineral density,
JOA hip score: Japanese Orthopaedic Association hip score
BMI: Body mass index

## References

[1] Shah N, Porter M (2005). Evolution of cemented stems. Orthopaedics.

[2] Bedard NA, Callaghan JJ, Stefl MD, Liu SS (2015). Systematic review of literature of cemented femoral components: what is the durability at minimum 20 years follow-up?. Clin Orthop Relat Res.

[3] Kazi HA, Whitehouse SL, Howell JR, Timperley AJ (2019). Not all cemented hips are the same: a register-based (NJR) comparison of taper-slip and composite beam femoral stems. Acta Orthop.

[4] Petheram TG, Whitehouse SL, Kazi HA (2016). The Exeter universal cemented femoral stem at 20 to 25 years: a report of 382 hips. Bone Joint J.

[5] Westerman RW, Whitehouse SL, Hubble MJW, Timperley AJ, Howell JR, Wilson MJ (2018). The Exeter V40 cemented femoral component at a minimum 10-year follow-up: the first 540 cases. Bone Joint J..

[6] Junnila M, Laaksonen I, Eskelinen A (2016). Implant survival of the most common cemented total hip devices from the Nordic arthroplasty register association database. Acta Orthop.

[7] Morita D, Iwase T, Ito T (2016). Bone resorption with cemented Exeter universal stem – three-years longitudinal DEXA study in 165 hips for femur. J Orthop Sci.

[8] Wroblewski BM, Siney PD, Fleming PA (2001). Triple taper polished cemented stem in total hip arthroplasty: rationale for the design, surgical technique, and 7 years of clinical experience. J Arthroplast.

[9] Buckland AJ, Dowsey MM, Stoney JD, Hardidge AJ, Ng KW, Choong PFM (2010). Periprosthetic bone remodeling using a triple-taper polished cemented stem in total hip arthroplasty. J Arthroplast.

[10] Ebramzadeh E, Sarmiento A, HA MK, Llinas A, Gogan W (1994). The cement mantle in total hip arthroplasty. analysis of long-term radiographic results. J Bone Joint Surg.

[11] Mulroy WF, Estok DM, Harris WH (1995). Total hip arthroplasty with use of so-called second-generation cementing techniques. a fifteen-year-average follow-up study. J Bone Joint Surg.

[12] Kawate K, Maloney WJ, Bragdon CR, SA SAB, Jasty M, Harris WH (1998). Importance of a thin cement mantle. Autopsy studies of eight hips. Clin Orthop Relat Res.

[13] Maloney WJ, Schmalzried T, Harris WH (2002). Analysis of long-term cemented total hip arthroplasty retrievals. Clin Orthop Relat Res.

[14] Langlais F, Kerboull M, Sedel L, Ling RSM (2003). The ‘French paradox.’. J Bone Joint Surg.

[15] El Masri F, Kerboull L, Kerboull M, Courpied JP, Hamadouche M (2010). Is the so-called 'French paradox' a reality?: long-term survival and migration of the Charnley-Kerboull stem cemented line-to-line. J Bone Joint Surg.

[16] Scheerlinck T, de Mey J, Deklerck R, Noble PC (2006). CT analysis of defects of the cement mantle and alignment of the stem: in vitro comparison of Charnley-Kerboull femoral hip implants inserted line-to-line and undersized in paired femora. J Bone Joint Surg.

[17] Skinner JA, Todo S, Taylor M, Wang JS, Pinskerova V, Scott G (2003). Should the cement mantle around the femoral component be thick or thin?. J Bone Joint Surg.

[18] Imura S (1995). Evaluation chart of hip joint functions. J Jpn Orthop Assoc.

[19] Barrack RL, Mulroy RD, Harris WH (1992). Improved cementing techniques and femoral component loosening in young patients with hip arthroplasty. A 12-year radiographic review. J Bone Joint Surg.

[20] Gruen TA, McNeice GM, Amstutz HC (1979). "modes of failure" of cemented stem-type femoral components: a radiographic analysis of loosening. Clin Orthop Relat Res.

[21] Harris WH, WA MG (1986). Loosening of the femoral component after use of the medullary-plug cementing technique. Follow-up note with a minimum five-year follow-up. J Bone Joint Surg.

[22] Fowler JL, Gie GA, Lee AJ, Ling RS (1988). Experience with the Exeter total hip replacement since 1970. Orthop Clin North Am.

[23] Purbach B, Kay PR, Siney PD, Fleming PA, Wroblewski BM (2013). The C-stem in clinical practice: fifteen-year follow-up of a triple tapered polished cemented stem. J Arthroplast.

[24] Young L, Duckett S, Dunn A (2009). The use of the cemented Exeter Universal femoral stem in a district general hospital: a minimum ten-year follow-up. J Bone Joint Surg.

[25] Schuroff AA, Deeke M, Pedroni MA, Lupselo FS, Rodrigo Kunz E, Lima AM (2017). Radiographic evaluation of cementation technique using polished, conical, triple-tapered femoral stem in hip arthroplasty. Rev Bras Ortop.

[26] Flatøy B, Röhrl SM, Rydinge J, Dahl J, Diep LM, Nordsletten L (2015). Triple taper stem design shows promising fixation and bone remodeling characteristics: radiostereometric analysis in a randomised controlled trial. Bone Joint J.

[27] Yates PJ, Burston BJ, Whitley E, Bannister GC (2008). Collarless polished tapered stem: clinical and radiological results at a minimum of ten years' follow-up. J Bone Joint Surg.

